# Minimal clinically important difference (MCID), patient-acceptable state (PASS), minimally detectable change (MDC), and substantial clinical benefit (SCB) in patients who have undergone arthroscopic surgery for femoroacetabular impingement: a systematic review

**DOI:** 10.1186/s10195-026-00951-5

**Published:** 2026-07-10

**Authors:** Filippo Migliorini, Nicola Maffulli, Luise Schäfer, Madhan Jeyaraman, Marcel Betsch, Mohamed Mahmoud

**Affiliations:** 1 Department of Trauma and Reconstructive Surgery, University Hospital of Halle, Martin-Luther University Halle-Wittenberg, Ernst-Grube-Street 40, 06097 Halle (Saale), Germany; 2 Department of Clinical Orthopaedics, Eifelklinik St.Brigida, 52052 Simmerath , Germany; 3 Department of Life Sciences, Health, and Health Professions, Link Campus University, Via del Casale Di San Pio V, 00165 Rome, Italy; 4 Department of Trauma and Orthopaedic Surgery, Faculty of Medicine and Psychology, University La Sapienza, 00185 Rome, Italy; 5 School of Pharmacy and Bioengineering, Faculty of Medicine, Keele University, Stoke on Trent, ST4 7QB UK; 6 Centre for Sports and Exercise Medicine, Barts and the London School of Medicine and Dentistry, Mile End Hospital,, Queen Mary University of London, London, E1 4DG UK; 7 Department of Orthopaedics, ACS Medical College and Hospital, Dr MGR Educational and Research Institute, Chennai, Tamil Nadu 600077 India; 8 Department of Trauma Surgery and Orthopaedics, University Hospital Erlangen, Friedrich-Alexander-University Erlangen-Nuremberg, 91054 Erlangen, Germany

**Keywords:** MCID, MDC, PASS, FAI, SCB, MIC PROMs

## Abstract

**Introduction:**

Hip arthroscopy has been established as a successful management for femoroacetabular impingement (FAI) syndrome. Patient-reported outcome measures (PROMs) are frequently used to assess treatment results in patients. However, clinically important outcome values have recently been calculated to better understand and interpret PROMs and define successful treatment. This systematic review aimed to investigate the minimal clinically important difference (MCID), patient-acceptable state (PASS), substantial clinical benefit (SCB), and minimally detectable change (MIC) of the most commonly used PROMs for assessing patients who have undergone arthroscopic surgery for FAI.

**Material and methods:**

This study was conducted according to the Preferred Reporting Items for Systematic Reviews and Meta-Analyses: the 2020 PRISMA statement. In October 2025, the following databases were accessed: PubMed, Web of Science, and Embase. All clinical studies investigating tools to assess the clinical relevance of PROMs used to evaluate patients who have undergone arthroscopic surgery for FAI were accessed. Only studies that assessed the minimal MCID, PASS, SCB, and/or MIC were considered.

**Results:**

The methodological quality of the 21 included studies was assessed, and the overall risk of bias was found to be low-to-moderate. The main characteristics of the included studies were analyzed and summarized. In addition, we determined the MCID, PASS, SCB, and MIC of the hip injury and osteoarthritis outcome score (HOOS), short form 36 (SF-36), modified Harris hip score (mHHS), nonarthritic hip score (NASH), Copenhagen hip and groin outcome score (HAGOS), hip outcome score (HOS), international hip outcome tool-12 (iHOT-12), international hip outcome tool-33 (iHOT-33), University of California, Los Angeles Score (UCLA), visual analogue scale (VAS), and Western Ontario and McMaster Universities Osteoarthritis Index (WOMAC) scores for arthroscopic hip surgery.

**Conclusions:**

This systematic review provides clinically important outcome values, such as the MCID, PASS, SCB, and MIC, for the most commonly used PROMs in arthroscopic hip surgery, based on data from 21 studies involving 5687 patients. The calculated values for the MCID, PASS, SCB, and MIC could be helpful for future clinical trials investigating the outcomes of hip arthroscopy in patients with FAI. However, given the heterogeneity of the included studies, their predominantly nonrandomized designs, and differences in PROMs, follow-up intervals, and calculation methods, these findings should be interpreted with caution.

*Level of evidence* Level IV, systematic review.

**Supplementary information:**

The online version contains supplementary material available at 10.1186/s10195-026-00951-5.

## Introduction

Femoroacetabular impingement (FAI) was coined by Smith-Petersen in 1936 [1, 2] and is characterized by an abnormal or problematic coupling or mismatch between the acetabulum, femoral head–neck junction, and femoral head with the loss of joint congruence [3, 4]. The overall incidence rate of FAI was reported to be 54.4 per 100,000 persons, with a higher incidence rate in females (73.2 per 100,000 persons) than in males (36.1 per 100,000 persons), which has increased over the last years [5, 6]. Clinically, FAI presents with unilateral intermittent groin/inguinal pain but may be bilateral, which becomes worse with continuous hip activity and hip flexion [7–9].

In 2001, Ganz and his coworkers introduced the classification of FAI [10, 11]. A distinction is made between three types: abnormal femur morphology is termed cam impingement, and abnormal morphology of the acetabulum is termed pincer impingement. If both types occur together, it is classified as mixed impingement [12–14]. Cam impingement occurs predominantly in young and middle-aged active males aged between 20 and 50 years with abnormal anterosuperior femoral head–neck junction, while pincer impingement occurs predominantly in young and middle-aged athletic females aged between 20 and 50 years with abnormal acetabular shape and/or orientation [15, 16].

Conservative measures such as rest, activity modification, physical therapy, and analgesic medication are recommended for the initial treatment of FAI [17–19]. Indications of surgery for FAI include pathological radiographic features combined with clinical findings, symptomatic FAI for longer than 3–6 months with failure of conservative measurements, and absence of hip osteoarthritis (OA) and severe articular damage [20–23]. Surgical techniques include arthroscopic or mini-open acetabular rim osteoplasty, or correction combined with labral repair, debridement, or refixation [24–28]. Periacetabular osteotomy (PAO) is used mainly for concomitant hip dysplasia and femoral osteochondroplasty [29].

Patient-reported outcome measures (PROMs) are reproducible, patient-related, and survey-based analytic tools that allow the assessment of different treatment types for various diseases/health problems in other patients and by different clinicians [30–32]. Over the last few years, the minimal clinically important difference (MCID), patient-acceptable symptom state (PASS), substantial clinical benefit (SCB), and minimally important change (MIC) have been increasingly used [33–35]. These tools aim to better understand the interpretation of clinically relevant values after surgical procedures and define what constitutes a successful procedure for a particular patient or a clinically significant change [36]. However, there is still a paucity in the literature regarding these clinically important outcome tools for commonly used PROMs in arthroscopic hip surgery, compounded by the considerable heterogeneity in how these measurements are calculated and their varied application across different outcome studies [37]. Although previous systematic reviews have addressed PROMs and clinically meaningful outcome thresholds in hip arthroscopy, the available evidence remains fragmented. Reported MCID, PASS, SCB, and MIC values vary considerably across PROMs instruments, patient cohorts, follow-up intervals, and calculation methods, limiting their direct comparability and clinical interpretation. Therefore, an updated synthesis focused specifically on the clinically relevant thresholds of commonly used PROMs after arthroscopic surgery for FAI is warranted. This systematic review aimed to investigate the MCID, PASS, SCB, and MIC of the most commonly used PROMs for assessing patients who have undergone arthroscopic surgery for FAI.

## Methods

### Eligibility criteria

All clinical studies investigating tools to assess the clinical relevance of PROMs used to evaluate patients who had undergone surgery for FAI were accessed. Eligible studies were required to report at least one of the following measures: MCID, PASS, SCB, or MIC. Only arthroscopic procedures and studies with levels I to IV of evidence according to the Oxford Centre of Evidence-Based Medicine [38], were considered. According to the authors’ language capabilities, articles published in English, German, Italian, French, or Spanish were considered. Reviews, opinions, letters, and editorials were excluded. Studies were also excluded if they did not report quantitative data for the outcomes of interest.

### Search strategy

This study was conducted according to the Preferred Reporting Items for Systematic Reviews and Meta-Analyses: the 2020 PRISMA statement [39]. The problem, intervention, comparison, outcomes, design (PICOD) algorithm was preliminarily established:

- P (problem): FAI;
- I (intervention): arthroscopic surgery;
- C (comparison): tool to assess the clinical efficacy of surgery;
- O (outcomes): MCID, PASS, SCB, MIC;
- D (design): clinical study.

The following databases were accessed in October 2025: PubMed, Web of Science, and Embase. No time constraint was set for the search. The Medical Subject Headings (MeSH) used for the database search are reported in the Appendix. No additional filters were used in the database search.

### Selection and data collection

Two authors (**; **) independently performed the database search. All the resulting titles were screened by hand, and the abstract was accessed if suitable. The full text of the abstracts that matched the topic was accessed. If the full text was not accessible or available, the article was not considered for inclusion. A cross reference of the bibliography of the full-text articles was also performed for inclusion. Disagreements were debated and mutually solved by the authors. A third author (**) made the final decision in the case of further disputes.

### Data items

Two authors (**; **) independently performed data extraction. The following data at baseline were extracted: author, year of publication, journal, country, number of patients, type of PROMs investigated, and type of analysis performed. Data on the MCID, PASS, SCB, and MIC were collected. The PROMs of interest were the hip injury and osteoarthritis outcome score (HOOS) [40] and its subscales, short form 36 (SF-36) [41–43], the modified Harris hip score (mHHS) [44, 45], nonarthritic hip score (NASH) [46], Copenhagen hip and groin outcome score (HAGOS) and related subscales [47], hip outcome score (HOS) and related subscales [48, 49], international hip outcome tool-12 (iHOT12) [50, 51], international hip outcome tool-33 (iHOT33) [52, 53], University of California, Los Angeles (UCLA) score [54], the visual analogue scale (VAS) [55], and Western Ontario and McMaster Universities osteoarthritis index (WOMAC) [56]. Data were extracted in Microsoft Office Excel version 16.72 (Microsoft Corporation, Redmond, USA).

### Methodological quality assessment and quality of the recommendations

The risk of bias was evaluated following the guidelines in the Cochrane Handbook for Systematic Reviews of Interventions [57]. Two reviewers (**; **) independently assessed the risk of bias in the extracted studies. Disagreements were solved by a third senior author (**). Given the lack of randomized controlled trials (RCTs), the risk of bias in nonrandomized studies of interventions (ROBINS-I) tool was used [58]. Seven domains of potential bias in non-RCTs were assessed. Possible confounding and the nature of patient selection before the comparative intervention are evaluated by two domains. A further domain is used to determine bias in the classification during the intervention. The final four domains assess the methodological quality after the intervention comparison has been implemented and relate to deviations from previously intended interventions, missing data, erroneous measurement of outcomes, and bias in the selection of reported outcomes. The ROBINS-I figure was elaborated using the Robvis Software (risk-of-bias visualization, Riskofbias.info, Bristol, UK) [59].

### Synthesis methods

The main author (**) performed the statistical analyses following the recommendations of the Cochrane Handbook for Systematic Reviews of Interventions [60]. For descriptive statistics, the arithmetic mean was used using International Business Machines SPSS software version 25 (IBM Corporation, Armonk, USA).

## Results

### Study selection

**Fig. 1:**
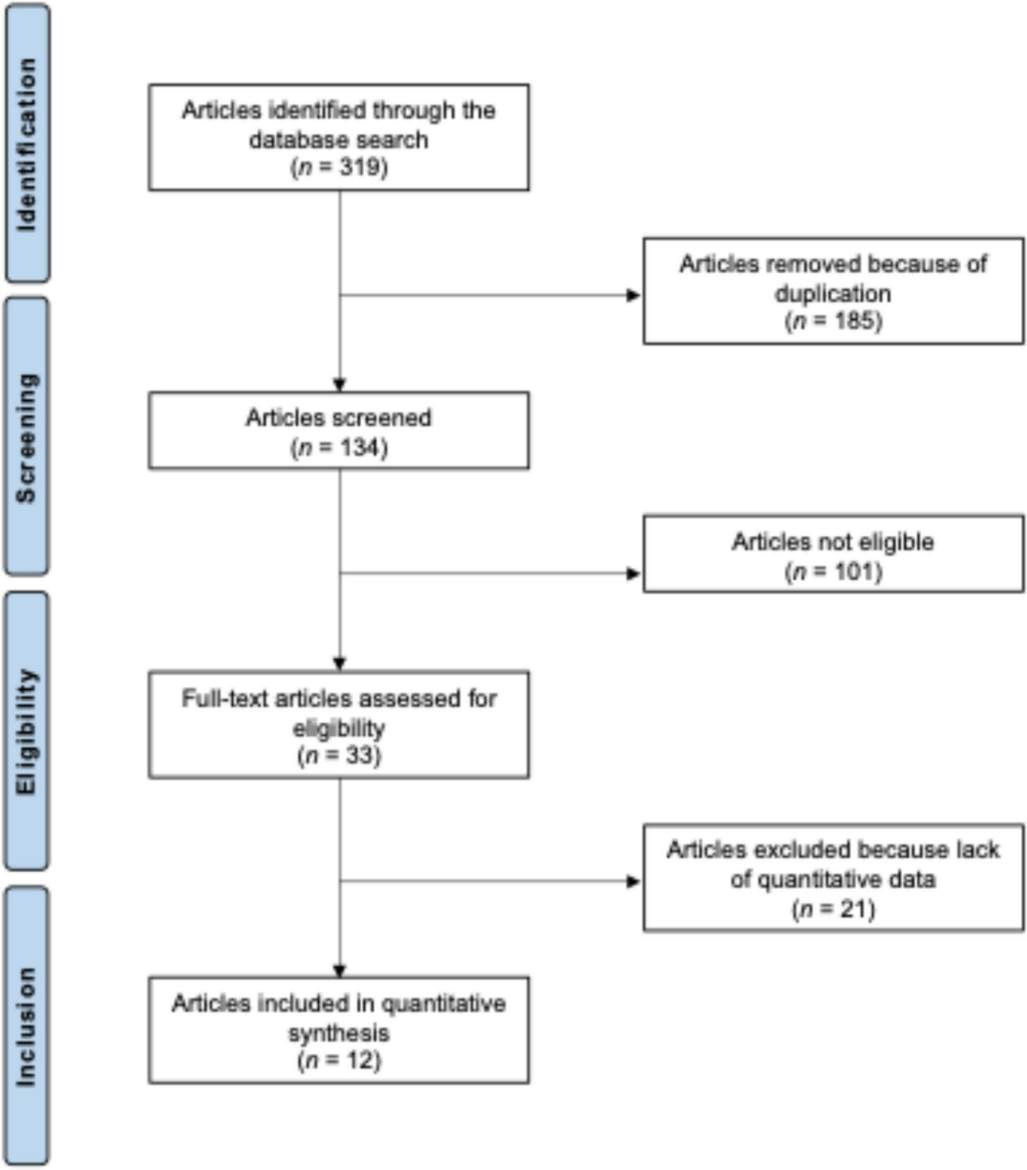
PRISMA flow chart of the literature search

A systematic literature search identified 921 clinical investigations that addressed the topic of interest. Of them, 285 studies were duplicates and were, therefore, excluded. The abstracts of the remaining 636 investigations were screened for eligibility. An additional 598 studies were discarded for lack of eligibility. In detail, the reasons for exclusion were inappropriate study type and design (*n* = 468), low level of evidence (*n* = 21), no arthroscopic procedures for FAI (*n* = 30), missing implementation of at least one tool to determine the clinical relevance of PROMs (MCID, PASS, SCB, or MIC) (*n* = 43), not reporting data from at least one PROM of interest (HOOS, SF-36, mHHS, NASH, HAGOS, HOS, iHOT12, iHOT33, UCLA, VAS, or WOMAC) (*n* = 19), and language limitations (*n* = 17). A further 17 studies did not include quantitative data on the end points of interest and were, therefore, not considered. This left 21 studies for final inclusion: 12 prospective and 9 retrospective clinical investigations. Although studies with levels I to IV of evidence were eligible, all included investigations were classified as level II or III; no level I or level IV study fulfilled all predefined eligibility criteria and provided extractable threshold data. The results of the literature search are shown in Fig. 1.

### Methodological quality assessment

**Fig. 2:**
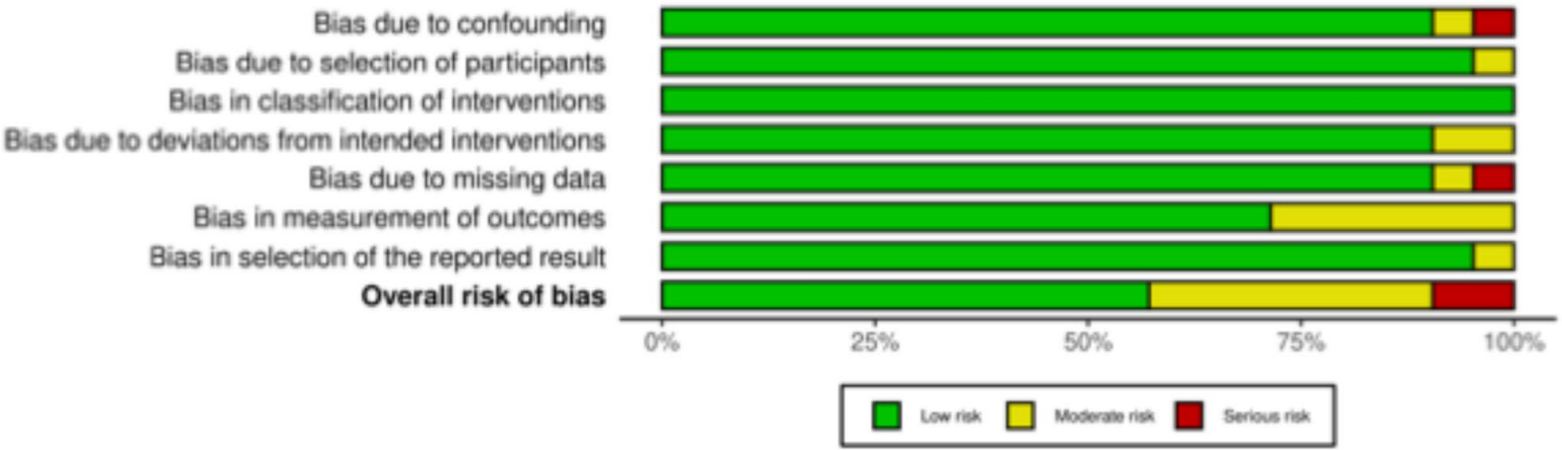
The ROBINS-I of non-RCTs

The ROBINS-I was applied to investigate the risk of bias in all studies in the present review. A total of 9.5% (2 of 21) of studies were rated as having a serious risk of bias in at least one domain but no critical risk in each domain. In most of the investigations, comparable intervention groups were formed at baseline, and intervention prediction was prevented by the influence of prognostic variables, leading to an overall low-to-moderate risk of bias from confounding. One study lacked a detailed description of the patient selection process. Exclusion of patients or differences in follow-up time of individual patients could not be detected, which indicates a predominantly low risk of bias from participant selection. The risk of bias in the intervention classification resulted uniformly low as neither nondifferential nor differential misclassification was identified. Bias during the intervention was mainly low and low-to-moderate in the domains assessed for risk of bias after the intervention. One study was identified with a serious risk of bias in the domain from missing data, but there was a low-to-moderate risk of bias in all other domains. Concerns in measuring outcomes arose from the lack of assessor blinding in all studies. Based on the ROBINS-I assessment, the overall risk of bias was low-to-moderate; however, this finding should be interpreted cautiously given the absence of randomized controlled trials and the lack of blinding across the included studies (Fig. 2).

### Study characteristics and results of individual studies

Data from 5687 patients were collected. The generalities and demographics of the included studies are shown in Table 1.

**Table 1:** Generalities and patient baseline of the included studies

Author	Year	Country	Level of evidence	Journal	Analysis	Type of PROMs	Patients (*n*)
Beck et al. [61]	2019	USA	III	*Am J Sports Med*	MCID/PASS/SCB	VAS	976
Beck et al. [62]	2021	USA	III	*Arthroscopy*	MCID/PASS	HOS/mHHS	300
Carton et al. [63]	2020	Ireland	III	*Orthop J Sports Med*	MCID	mHHS/SF-36	576
Chahal et al. [64]	2014	Canada, USA	II	*Orthop J Sports Med*	MCID/PASS	mHHS/HOS	130
Domb et al. [65]	2023	USA	II	*J Bone Joint Surg Am*	MCID	mHHS/NASH/HOS	45
Huang et al. [66]	2022	China	III	*Arthroscopy*	MCID/PASS	mHHS	159
Kemp et al. [67]	2013	Australia	II	*Am J Sports Med*	MIC	HAGOS/HOOS/HOS/iHOT-33/mHHS	45
Litrenta et al. [68]	2020	USA	III	*J Ped Orthop*	MCID/PASS	mHHS	81
Martin et al. [49]	2008	USA	II	*Arthroscopy*	MCID	HOS	126
Martin et al. [69]	2018	USA	III	*Arthroscopy*	MCID/SCB	iHOT-12	577
Menge et al. [70]	2021	USA	III	*Am J Sports Med*	MCID/PASS	mHHS/HOS	60
Mullins et al. [71]	2021	Ireland	II	*Orthop J Sports Med*	MCID	mHHS/UCLA/SF-36/WOMAC	1171
Mullins et al. [72]	2023	Ireland	III	*Knee Surg Sports Traumatol Arthrosc*	MCID	mHHS/UCLA/SF-36/WOMAC	97
Nwachukwu et al. [73]	2017	USA	II	*Am J Sports Med*	MCID	HOS/iHOT-33/mHHS	364
Nwachukwu et al. [74]	2017	USA	II	*Arthroscopy*	MCID/SCB	mHHS/HOS/iHOT-33	47
Nwachukwu et al. [75]	2018	USA	III	*Arthroscopy*	MCID/SCB	mHHS/HOS/iHOT-33	49
Nwachukwu et al. [76]	2020	USA	II	*Am J Sports Med*	MCID/SCB/PASS	HOS/iHOT-12/mHHS	283
Philippon et al. [77]	2020	USA	III	*J Bone Joint Surg*	MCID/PASS	HOS/mHHS	89
Saks et al. [78]	2022	USA	II	*Arthroscopy*	MCID/PASS	mHHS/NASH/iHOT-12/VAS	82
Serong et al. [79]	2022	Germany	II	*J Clin Med*	MCID/PASS	iHOT-33	353
Thorborg et al. [80]	2018	Denmark	II	*Am J Sports Med*	MIC	HAGOS/mHHS	77

*PROMs* patient-reported outcome measures, *MCID* minimal clinically important difference, *PASS* patient acceptable symptom state, *SCB* substantial clinical benefit, *MIC* minimally important change, *HOOS* hip injury and osteoarthritis outcome score, *SF* short form, *mHHS* modified Harris hip score, *NASH* nonarthritic hip score, *HAGOS* Copenhagen hip and groin outcome score, *HOS* hip outcome score, *iHOT* international hip outcome tool, *UCLA* University of California, Los Angeles, *VAS* visual analogue scale, *WOMAC* Western Ontario and McMaster Universities osteoarthritis index

### Results syntheses

Table 2 reports an overview of the results of MCID, PASS, SCB, and MIC regarding the HOOS, SF-36, mHHS, NASH, HAGOS, HOS, iHOT-12, iHOT-33 UCLA, VAS, and WOMAC.

**Table 2:** Main results

PROMs	Patients (*n*)	MCID	PASS	SCB	MIC
HAGOS ADL	167				9.0
HAGOS pain	77				9.1
HAGOS participation in physical activities	77				12.1
HAGOS physical activity	90				1.0
HAGOS quality of life	167				9.0
HAGOS sport & recreation	167				9.0
HAGOS symptoms	77				8.4
HOOS ADL	90				6.0
HOOS pain	90				9.0
HOOS QoL	90				11.0
HOOS sport & recreation	90				10.0
HOOS symptoms	90				9.0
HOS ADL	1338	14.8	75.2	96.6	5.0
HOS sports & recreation	1383	19.9	68.7	91.4	6.0
iHOT-12	942	13.0	74.3	57.8	
iHOT-33	903	25.1	66.2	85.9	10.0
mHHS	3817	14.6	70.7	94.0	8.0
NASH	127	8.8			
SF-36	1844	15.0			
UCLA	1268	0.8			
VAS	1021	14.8	21.6	15.4	
WOMAC	1268	7.0			

*PROMs* patient-reported outcome measures, *MCID* minimal clinically important difference, *PASS* patient acceptable symptom state, *SCB* substantial clinical benefit, *MIC* minimally important change, *HOOS* hip injury and osteoarthritis outcome score, *SF* short form; *mHHS* modified Harris hip score, *NASH* nonarthritic hip score, *HAGOS* Copenhagen hip and groin outcome score, *HOS* hip outcome score, *iHOT* international hip outcome tool, *UCLA* University of California, Los Angeles, *VAS* visual analogue scale, *WOMAC* Western Ontario and McMaster Universities osteoarthritis index, *ADL* activity of day living, *QoL* quality of life

## Discussion

This systematic review examined the most commonly used clinically important outcome values in hip arthroscopy (MCID, PASS, SCB, and MIC), including the HOOS, SF-36, mHHS, NASH, HAGOS, HOS, iHOT12, iHOT33, UCLA Score, VAS, and the WOMAC scores. In recent years, clinically important outcome values have become of great interest as they provide threshold information on absolute or changing values scores related to the functional status of the patient, thus allowing assessment of surgical success [81–83]. Since there is no consensus for using a single PROM in the management of FAI, we chose to establish relevant outcome values in the most commonly used scores. Despite differences in the treatment cohorts, follow-up time points, and methodology of the included studies, we could establish and summarize the range of clinically relevant outcome measures (Table 2).

Over the last few years, FAI has gained greater clinical interest since it may lead to premature hip osteoarthritis if left untreated [84]. The reported causes of FAI include morphological anomalies such as retroversion of the acetabulum, reduced anteversion in flexion and internal rotation, increased anteversion in external rotation and reduced inclination of the acetabulum [85, 86]. Increased acetabular depth, overlap of the femur, asphericity of the femoral head (coxa recta), and reduced head–neck offset may favor FAIs both genetically and owing to repetitive exercise during musculoskeletal maturation [87–89]. Traumatic events, such as prior hip surgeries (e.g., femoral/acetabular osteotomies) or fracture sequelae (e.g., misaligned femoral neck fractures), as well as developmental pathologies (e.g., epiphyseal malalignment, Legg–Calve–Perthes disease, or coxa vara), can contribute to FAI [90–92]. Morphological abnormalities of the acetabulum and proximal femur may impair the anterosuperior hip joint, causing gradual femoral head approximation towards the acetabulum [93]. This leads to femoral neck impact against the acetabular rim, resulting in cartilage and labral damage, progressive joint degeneration, and premature hip osteoarthritis, with the anterolateral cartilage being the most affected [84, 94, 95].

Recently, clinically important outcome values have been established to transform PROMs into clinically relevant results [96, 97]. One of the most commonly used measures is the MCID, the smallest change in a PROM, from baseline to follow-up, that leads to a meaningful improvement for the patient [98, 99]. Another clinically important outcome measure is the PASS, consisting of a simple “yes” or “no” answer about the patient’s satisfaction, which is considered the highest level of symptom past which patients consider themselves well [100, 101]. SCB is a significant improvement in patient-reported outcomes, representing a higher threshold of clinical relevance than the MCID. In the scientific literature, SCB is conceptualized in two distinct ways: as a quantified change score from baseline or as a threshold value that, when exceeded, indicates the attainment of SCB. These differing interpretations underscore the necessity for standardized definitions and methodologies in their application across clinical studies [75, 102]. Lastly, the MIC is the smallest change in a PROM, essential to the patient [103].

A growing number of PROMs are being considered for the management of FAI [104, 105]. However, this vast number limits the ability to compare PROMs quantitatively across studies. Therefore, efforts have been undertaken to standardize the use of PROMs in clinical studies, such as the patient-reported outcomes measurement information system (PROMIS), which uses automated adaptive testing to help compare treatment options in various anatomical locations [106, 107]. Most included studies (95.2%, 20 of 21) reported the recorded values of different analyses for different PROMs types, significant clinical improvement after hip arthroscopy for FAI, and recommended hip arthroscopy as a beneficial surgical intervention for FAI in both athletic and nonathlete populations. Most studies reported a high or complete level of patient satisfaction. In the study by Carton et al., a significant difference was found in the changes in the MCID score when comparing baseline patient data using the average score change in the mHHS and SF36 [63]. However, when the patient-oriented proportional index (POPI) technique was used, no difference was observed in the scores relative to the baseline.

The ceiling effect of a PROM is described as a limitation or restriction of the targeted score, which occurs when a large section of the patients give the highest/best score, leading to the accumulation of PROM values/scores at or near the upper limit of the related scale, making it inaccurate, misleading, less expressive, and less discriminative [108, 109]. A ceiling effect of a PROM can emerge from the structure of the questions and how the patients understood and interpreted them [108]. Two studies [63, 71] (9.52%, 2 of 21) reported the influence of the ceiling effect on the recorded values, which makes the scores inaccurate and risky to interpret. Both recommended using the POPI method in the evaluation (e.g., investigation of MCID) [63, 71]. However, the ceiling effect was negated in one study and was not mentioned in the other studies, neither absent nor present.

In the included studies, clinically relevant thresholds were not calculated using a uniform methodological approach. Different studies applied anchor-based, distribution-based, receiver operating characteristic, or proportional methods, including the patient-oriented proportional index [63, 71, 96, 98, 99, 103]. These approaches reflect different statistical and clinical concepts and may, therefore, yield different threshold values for the same PROM. For example, anchor-based methods relate PROM changes to an external patient-perceived reference, whereas distribution-based methods are more strongly driven by the statistical properties of the sample [98, 99, 103]. This methodological variability may partly explain the differences observed across MCID, PASS, SCB, and MIC values and should be considered when interpreting the thresholds summarized in this review.

From a clinical perspective, the threshold values summarized in this review may help clinicians and researchers interpret whether postoperative changes in PROMs are likely to be meaningful to patients rather than merely statistically detectable. However, these values should not be applied as universal cut-offs for all patients undergoing hip arthroscopy. Their interpretation should consider the PROM used, baseline symptom severity, follow-up duration, patient expectations, activity level, and the method used to calculate the threshold.

Some limitations need to be addressed when interpreting the results of this systematic review as it includes multiple studies which are heterogeneous and differ in many aspects such as level of evidence, study design (case series, cohorts, etc.), participants (age, sex, activity, BMI, etc.), controlled or noncontrolled, single or multicentered study, size of cohorts (small-, medium-, and large-sized studies), number and time points of the assessments during the follow-ups, PROMs types, and numbers and types of the analyses. Although studies with levels I to IV of evidence were eligible, the final evidence base consisted only of level II and III investigations. The absence of level I evidence limits the strength of the conclusions, although this reflects the available literature meeting the predefined eligibility criteria rather than a restriction imposed during study selection. In addition, there were differences in the eligibility criteria, surgical techniques, postoperative rehabilitation, and concomitant procedures performed between the studies. These differences were not considered in the analysis of the findings, but they could profoundly affect the results. We also did not adjust our conclusions on the basis of differences in the methods used to calculate the outcomes of interest (anchor- versus distribution-based methods). Also, many studies used few numbers/types of PROMs and some analyses were not investigated for many PROMs. MCID, PASS, and SCB were not measured for all HAGOS and HOOS subscales, and PASS, SCB, and MIC were not investigated for NASH, SF-36, UCLA, and WOMAC, further limiting the results of this study. The reported values represent a simple arithmetic mean of the data extracted from the existing literature rather than a pooled estimate derived through formal statistical methods. Given the heterogeneity in study methodologies, patient populations, and statistical approaches, a meta-analytic aggregation was not performed, and no weighting was applied on the basis of sample size or variance. Although no time restriction was applied to maximize the sensitivity of the search, this may have contributed to methodological heterogeneity, as studies published in different periods may differ in PROM selection, follow-up protocols, surgical techniques, rehabilitation strategies, and threshold calculation methods. Finally, the language restriction to English, German, Italian, French, and Spanish represents an additional limitation, as relevant studies published in other languages may have been missed. This may introduce language-related selection bias and could limit the cultural and international generalizability of the findings, particularly because PROM interpretation and clinically meaningful threshold values may be influenced by linguistic, cultural, and population-related differences. Consequently, the reported averages should be interpreted cautiously, as they do not account for variability in study quality, methodological differences, or potential biases inherent in the original studies. Additionally, without formal heterogeneity assessments (e.g., *I*^2^ statistics or Cochran’s *Q* test), the variability among studies remains unquantified. The summarized threshold values, the number of contributing patients, and the characteristics of the included studies are provided to ensure transparency and to support interpretation of the reported findings. Future research using more standardized methodologies and meta-analytic techniques may be warranted to derive more precise estimates.

## Conclusions

This systematic review provides clinically relevant outcome values, such as the MCID, PASS, SCB, and MIC, for the most commonly used PROMs in arthroscopic hip surgery using data from 21 studies with a total of 5687 patients. The MCID, PASS, SCB, and MIC calculated values are valuable for future hip arthroscopy trials in patients with FAI. However, given the heterogeneity of the included studies, their predominantly nonrandomized design, and differences in PROMs, follow-up intervals, and calculation methods, these findings should be interpreted cautiously. These metrics should ideally be calculated in a cohort-specific manner, as relying on prepublished values or summative scores from different studies may not accurately reflect future trial outcomes.

## Supplementary information

Supplementary material 1.

## Data Availability

The datasets generated during and/or analyzed during the current study are available throughout the manuscript.

## Abbreviations

ADL: Activity of daily living
FAI: Femoroacetabular impingement
HAGOS: Copenhagen hip and groin outcome score
HOOS: Hip injury and osteoarthritis outcome score
HOS: Hip outcome score
iHOT-12: International hip outcome tool-12
iHOT-33: International hip outcome tool-33
MCID: Minimal clinically important difference
MeSH: Medical subject headings
mHHS: Modified Harris hip score
MDC: Minimally detectable change
MIC: Minimally important change
NASH: Nonarthritic hip score
OA: Osteoarthritis
PAO: Periacetabular osteotomy
PASS: Patient-acceptable symptom state
PICOD: Problem, intervention, comparison, outcomes, design
POPI: Patient-oriented proportional index
PRISMA: Preferred reporting items for systematic reviews and meta-analyses
PROMIS: Patient-reported outcomes measurement information system
PROMs: Patient-reported outcome measures
QoL: Quality of life
RCTs: Randomized controlled trials
ROBINS-I: Risk of bias in nonrandomized studies of interventions
SCB: Substantial clinical benefit
SF-36: Short form 36
UCLA: University of California, Los Angeles Score
VAS: Visual analogue scale
WOMAC: Western Ontario and McMaster Universities Osteoarthritis Index
