# Supplementary material for: Minimal clinically important difference (MCID), patient-acceptable state (PASS), minimally detectable change (MDC), and substantial clinical benefit (SCB) in patients who have undergone arthroscopic surgery for femoroacetabular impingement: a systematic review
Source: J Orthop Traumatol. 2026 Jul 10;27:34. doi: 10.1186/s10195-026-00951-5 (PMC13356118; doi:10.1186/s10195-026-00951-5)
Supplement: Supplementary file 1 — Supplementary material 1. [file 10195_2026_951_MOESM1_ESM.docx]

**Systematic literature search for:**

**Minimal clinically important difference (MCID), patient-acceptable symptom state (PASS), minimally detectable change (MDC), clinically important difference (CID), and substantial clinical benefit (SCB) in patients who have undergone arthroscopic surgery for femoroacetabular impingement: a systematic review**

**Concept 1: femoroacetabular impingement**

**Keywords:**

femoroacetabular impingement

FAI

**Mesh:**

"Femoracetabular Impingement"[Mesh]

"Femoracetabular Impingement/surgery"[Mesh]

**Concept 2: arthroscopic surgery**

**Keywords:**

arthroscopic surgery

**Mesh:**

"Arthroplasty"[Mesh]

"Arthroplasty, Replacement, Hip"[Mesh]

**Concept 3: Interest in Outcome**

**Keywords**

**clinical efficacy of surgery**

minimal clinically important difference (MCID)

patient-acceptable symptom state (PASS)

minimally detectable change (MDC)

clinically important difference (CID)

substantial clinical benefit (SCB)

**Mesh:**

"Treatment Outcome"[Mesh]

"Minimal Clinically Important Difference"[Mesh]

**PROM**

Hip Injury and Osteoarthritis Outcome Score (HOOS)

Short Form 36 (SF-36)

modified Harris Hip Score (mHHS) Nonarthritic Hip Score (NASH) Copenhagen Hip and Groin Outcome Score (HAGOS)

Hip Outcome Score (HOS)

International Hip Outcome Tool-12 (iHOT12)

International Hip Outcome Tool-33 (iHOT33)

University of California Los Angeles (UCLA) Score

Visual analogue scale (VAS)

Western Ontario and McMaster Universities Osteoarthritis (WOMAC)

**Mesh:**

"Treatment Outcome"[Mesh]

"Patient Outcome Assessment"[Mesh]

"Patient Reported Outcome Measures"[Mesh]

"Visual Analog Scale"[Mesh]

**Concept 1:**

"Femoracetabular Impingement"[Mesh] OR "Femoracetabular Impingement/surgery"[Mesh] OR femoroacetabular impingement OR FAI

**AND**

**Concept 2:**

"Arthroplasty"[Mesh] OR "Arthroplasty, Replacement, Hip"[Mesh] OR arthroscopic surgery

**AND**

**Concept 3:**

"Treatment Outcome"[Mesh] OR "Minimal Clinically Important Difference"[Mesh] OR clinical efficacy of surgery OR minimal clinically important difference OR MCID OR patient-acceptable symptom state OR PASS OR minimally detectable change OR MDC OR clinically important difference OR CID OR substantial clinical benefit OR SCB

**AND**

"Patient Outcome Assessment"[Mesh] OR "Patient Reported Outcome Measures"[Mesh] OR "Visual Analog Scale"[Mesh] OR PROM OR Hip Injury and Osteoarthritis Outcome Score OR HOOS OR Short Form 36 OR SF-36 OR modified Harris Hip Score OR mHHS OR Nonarthritic Hip Score OR NASH OR Copenhagen Hip and Groin Outcome Score OR HAGOS OR Hip Outcome Score OR HOS OR International Hip Outcome Tool-12 OR iHOT12 OR International Hip Outcome Tool-33 OR iHOT33 OR University of California Los Angeles Score OR UCLA OR Visual analogue scale OR VAS OR Western Ontario and McMaster Universities Osteoarthritis OR WOMAC

**Summary of the Search**

((("Femoracetabular Impingement"[Mesh] OR "Femoracetabular Impingement/surgery"[Mesh] OR femoroacetabular impingement OR FAI) AND ("Arthroplasty"[Mesh] OR "Arthroplasty, Replacement, Hip"[Mesh] OR arthroscopic surgery)) AND ("Treatment Outcome"[Mesh] OR "Minimal Clinically Important Difference"[Mesh] OR clinical efficacy of surgery OR minimal clinically important difference OR MCID OR patient-acceptable symptom state OR PASS OR minimally detectable change OR MDC OR clinically important difference OR CID OR substantial clinical benefit OR SCB)) AND ("Patient Outcome Assessment"[Mesh] OR "Patient Reported Outcome Measures"[Mesh] OR "Visual Analog Scale"[Mesh] OR PROM OR Hip Injury and Osteoarthritis Outcome Score OR HOOS OR Short Form 36 OR SF-36 OR modified Harris Hip Score OR mHHS OR Nonarthritic Hip Score OR NASH OR Copenhagen Hip and Groin Outcome Score OR HAGOS OR Hip Outcome Score OR HOS OR International Hip Outcome Tool-12 OR iHOT12 OR International Hip Outcome Tool-33 OR iHOT33 OR University of California Los Angeles Score OR UCLA OR Visual analogue scale OR VAS OR Western Ontario and McMaster Universities Osteoarthritis OR WOMAC)
